# Geostatistical analysis for spatial distribution of anemia (Hb level) among women of reproductive age and determinant factors

**DOI:** 10.1002/fsn3.3408

**Published:** 2023-06-11

**Authors:** Ghulam Abbas, Azhar Hussain, Abid Hussain, Zahoor Ahmed, Yasir Abbas, Arash Nemat

**Affiliations:** ^1^ Department of Biological Sciences Karakoram International University Gilgit Pakistan; ^2^ Department of Agriculture and Food Technology Karakoram International University Gilgit Pakistan; ^3^ School of Food & Biological Engineering Jiangsu University Zhenjiang China; ^4^ Department of Human Nutrition and Dietetics, School of Food and Agricultural Sciences University of Management and Technology Lahore Lahore Pakistan; ^5^ Kabul University of Medical Sciences Kabul Afghanistan

**Keywords:** anemia, district Gilgit, GIS mapping reproductive age group, women

## Abstract

The study was designed to assess the geostatistical spatial distribution of anemia and determinant factors among the women of reproductive age group (RAG) in Gilgit district, Pakistan. The Hb levels for 15–25 RAG, 26–35 RAG, and 36–45 group showed 10.22 g/dL, 10.41 g/dL, and 9.90 g/dL levels, respectively, while the Hb level showed a nugget/sill ratio of 0.21 inferring strong for the 15–25 group, weak for the 26–36, and moderate for 36–45 spatial dependence. Furthermore, 15–25 RAG showed 8% severe and 33.34% sufficient cases and 26–35 showed 12% severe and 29.33% sufficient results in their Hb level, whereas 36–45 had 9.34% severe and 29.33% sufficient.

## Graphical abstract

The key determinant factors were education, diet, age, BMI, latrine facilities, improved and unimproved water sources, diarrhea, intestinal worms, chronic disease, family type, and unemployment in the study. Among education, diet, BMI, latrine facilities, unimproved water source, intestinal worms, diarrhea, chronic disease, family type, unemployment, lactating women, pregnant women, and excessive use of tea were the triggering factors. To cope with this issue, awareness sessions, workshops, and seminars must be conducted in Gilgit district.

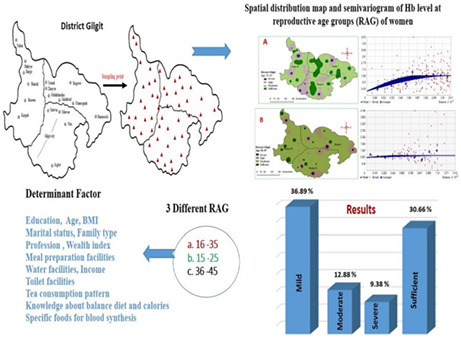

## 1 INTRODUCTION

Anemia is a state of hemoglobin (Hb) level less than normal, responsible for adverse health, lost productivity, and premature death. Surveys showed that one‐third world's population is reported to suffer from anemia and its epidemiology varies based on age, geographical region, culture, and sex (Varghese & Stein, 2019; Yoshimura et al., 2021) (Zulfiqar et al., 2021). Moreover, women of reproductive age are more susceptible to anemia due to menstrual blood loss and pregnancy (Kinyoki et al., 2021). The prevalence of anemia is highest in South Asian countries across all age groups (Chaparro & Suchdev, 2019). Similarly, anemia among women with RAGs ranges from moderate to severe and has also been reported in developing countries.

The major determinant factors studied are poverty, poor dietary conditions, high burden of diseases, illiteracy, and others. Moreover, undernutrition, inadequate dietary intake during pregnancy, poor water hygiene, sanitation status, rural residency, and parasitic infections are the other major factors responsible for iron deficiency (Chaparro & Suchdev, 2019; Kinyoki et al., 2021).

Among the developing countries, Pakistan has been specified at risk of iron, iodine, and zinc deficiencies. According to a recent National Nutritional Survey (MNH, 2018), about 41.7% of women of reproductive age are anemic with a slightly higher proportion in rural (44.3%) than in urban settings (40.2%), while WHO Global reported Pakistan's proportion of the population with anemia up to 50.9% a severe condition. Furthermore, WHO recommended a target of a 50% reduction of anemia among women of reproductive age by 2025 (WHO, 2014). Therefore, the study was designed to achieve the WHO global nutrition target 2025 and to explore major factors of anemia, useful in timely interventions in its prevention across the region among women of RAGs.

## 2 MATERIALS AND METHODS

**FIGURE 1:**
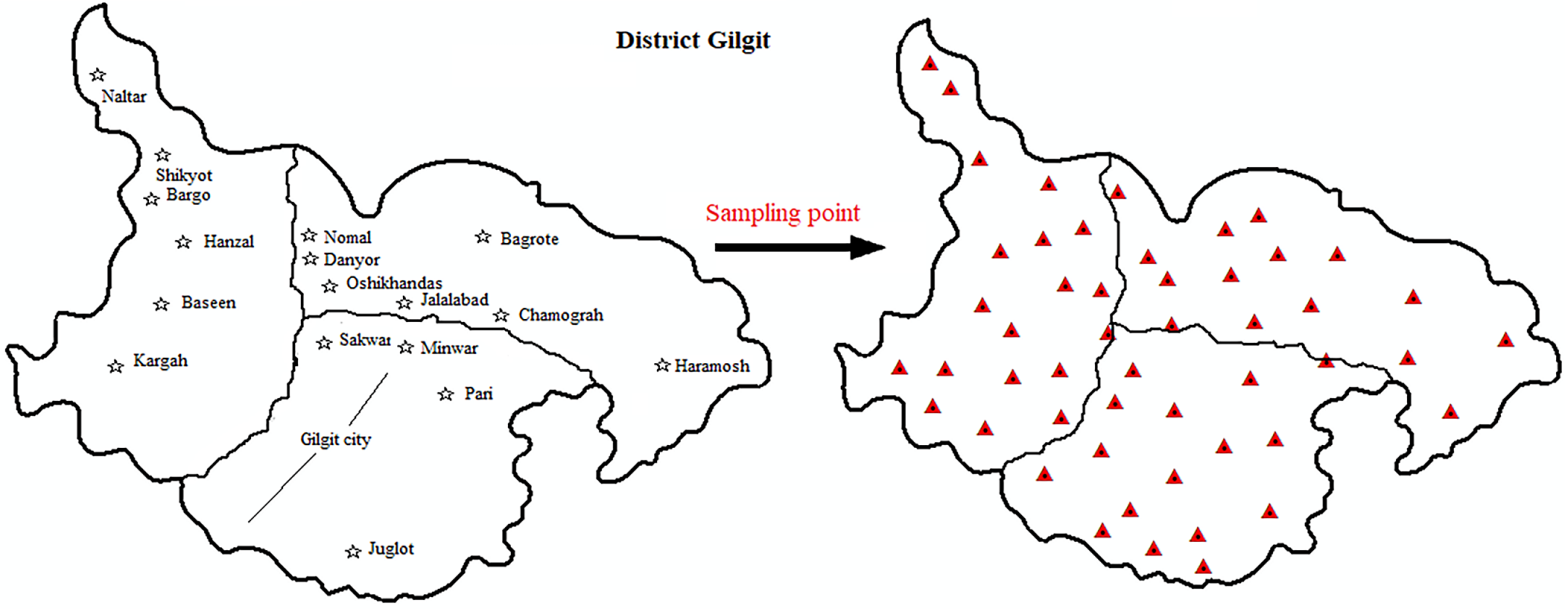
Map of the study area and sampling points.

The current study was conducted in Gilgit district and divided into three strata (I, II, and III). A total of 675 women with three RAGs (15–25, 26–35, and 36–45) were randomly selected from three strata of the district. Within each stratum 225 and each RAG of 75 women were selected (Figure 1).

### 2.1 Anemia (Hb level) analysis

At the time of enrollment, 10 μL of blood was collected as described by standard procedures. The hemoglobin concentration was determined by a handheld instrument, called a hemoglobinometer.

### 2.2 Statistical analysis

Statista 8.1 software was used to assess, descriptive statistics, and analysis of variance.

### 2.3 Geostatistical analysis and mapping

Geostatistical analysis was employed to describe the spatial distribution of Hb level in women of RAG. ArcGIS 10.4 and ArcGIS Geostatistical Analyst were exploited for mapping of Hb level. The kriging technique was used to interpolate the values of unsampled locations as described by Caers (2003). The following formula was used for kriging;

$$\overset{N}{\underset{i=1}{\hat{Z}\left(s_{0}\right)=\sum N\;\hat{Z}\;\lambda_{i}\;Z\left(s_{i}\right)}}$$

Where *Z* (*s*_*i*_) is the measured value at the *i*th location, *λ*_*i*_ is an unknown weight for the measured value at the *i*th location, *s*_0_ is the prediction location, and *N* is the number of measured values.

Semivariogram can be fitted with a spherical model, presented by (Olea, 2012);

$$\text{Spherical}=y\left(h\right)=\left\{C_{0}+C\;\left(3h/2\mathrm{ac}\right)–½{\left(h/a\right)}^{3}\;\begin{array}{c} 0\leq h<a\\ h\geq a \end{array}\right.$$

Spatial dependence (SDP %) was calculated as described by Biondi et al. (1994) and is given by the following expression;

$$\mathrm{SDP}\;\text{Spherical}\%=\frac{C_{1}}{C_{0}+C_{1}}\times 100$$

For the spherical semivariogram: SDP Spherical (%); ≤2.5% strong spatial dependence; 25% <SPD (%) ≤75% moderate spatial dependence, and ≥75% weak spatial dependence. The spatial analyst function of Arc GIS software was used to prepare maps as described by Hussain et al. (2019).

### 2.4 Determinant factor of anemia

**FIGURE 2:**
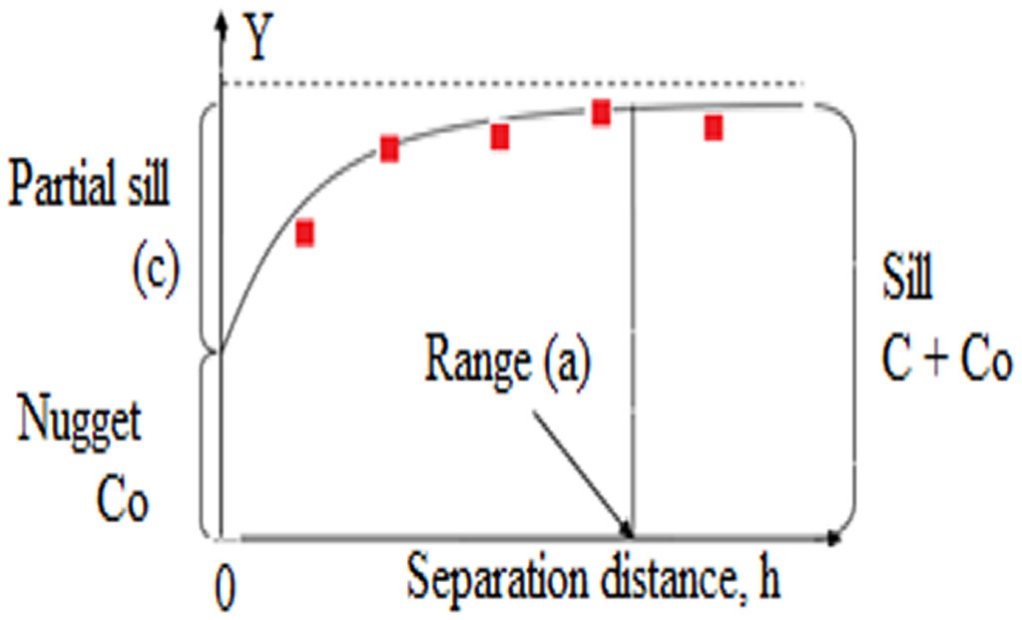
Spherical model used in the study.

**FIGURE 3:**
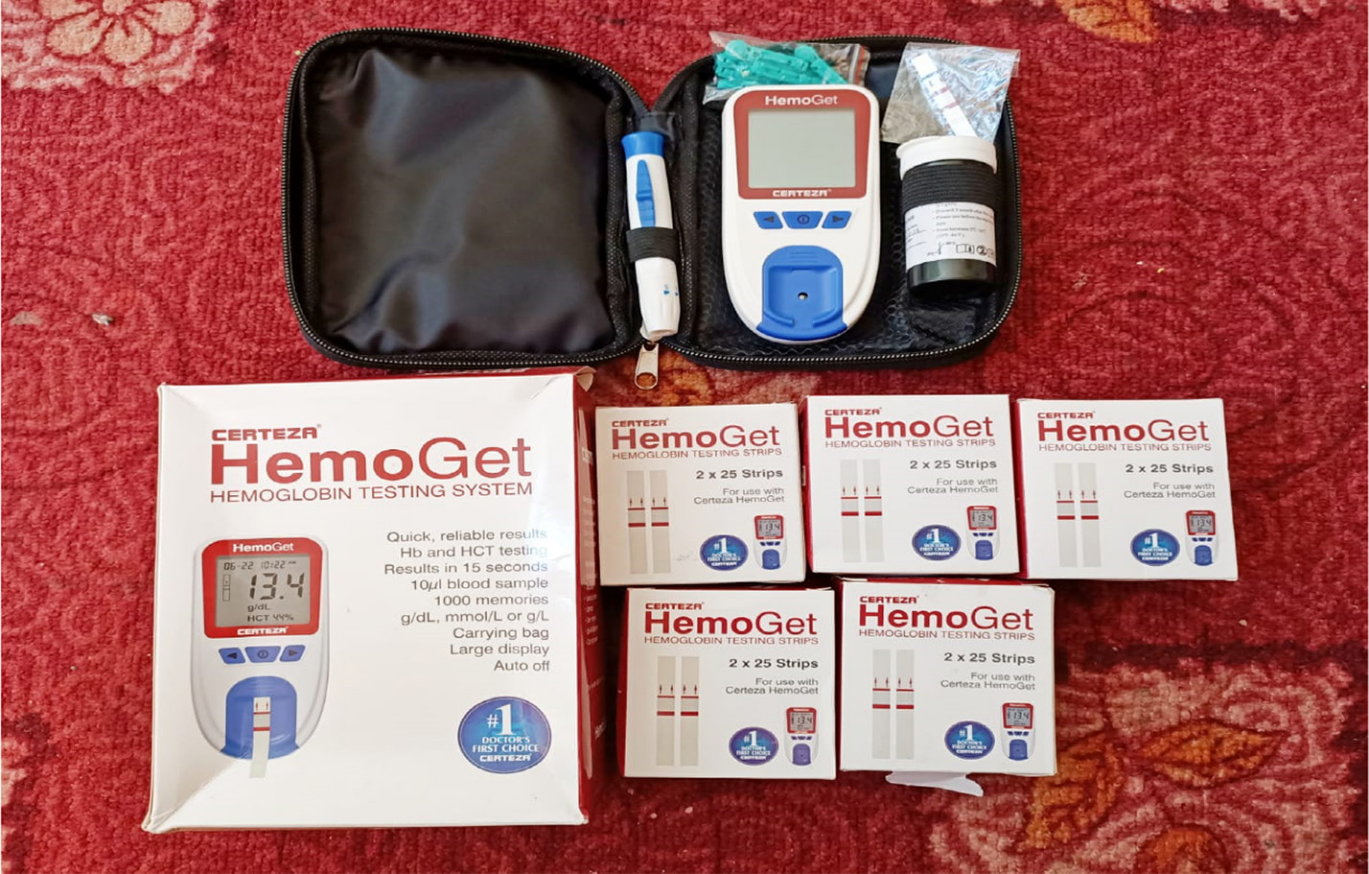
HemoGet Hemoglobin testing system (Certeza).

This study was based on primary data collected from women of different RAG in Gilgit district during the year August 2020 to March 2021. A sample survey was undertaken and personal interviews were conducted to collect information about determinant factors of anemia. HemoGet Hemoglobin testing system (Certeza) was used for the anemia estimations in the study (Figures 2 and 3).

### 2.5 Sample size

The survey was based on 120 women of RAG randomly selected from the study area. Forty women of each reproductive age were selected. The analysis was carried out concerning percentage analysis by using the following formula:

Frequency % = No of responses from each question/Total number of respondents ×100.

## 3 RESULTS AND DISCUSSION

### 3.1 Descriptive statistical analysis

**FIGURE 4:**
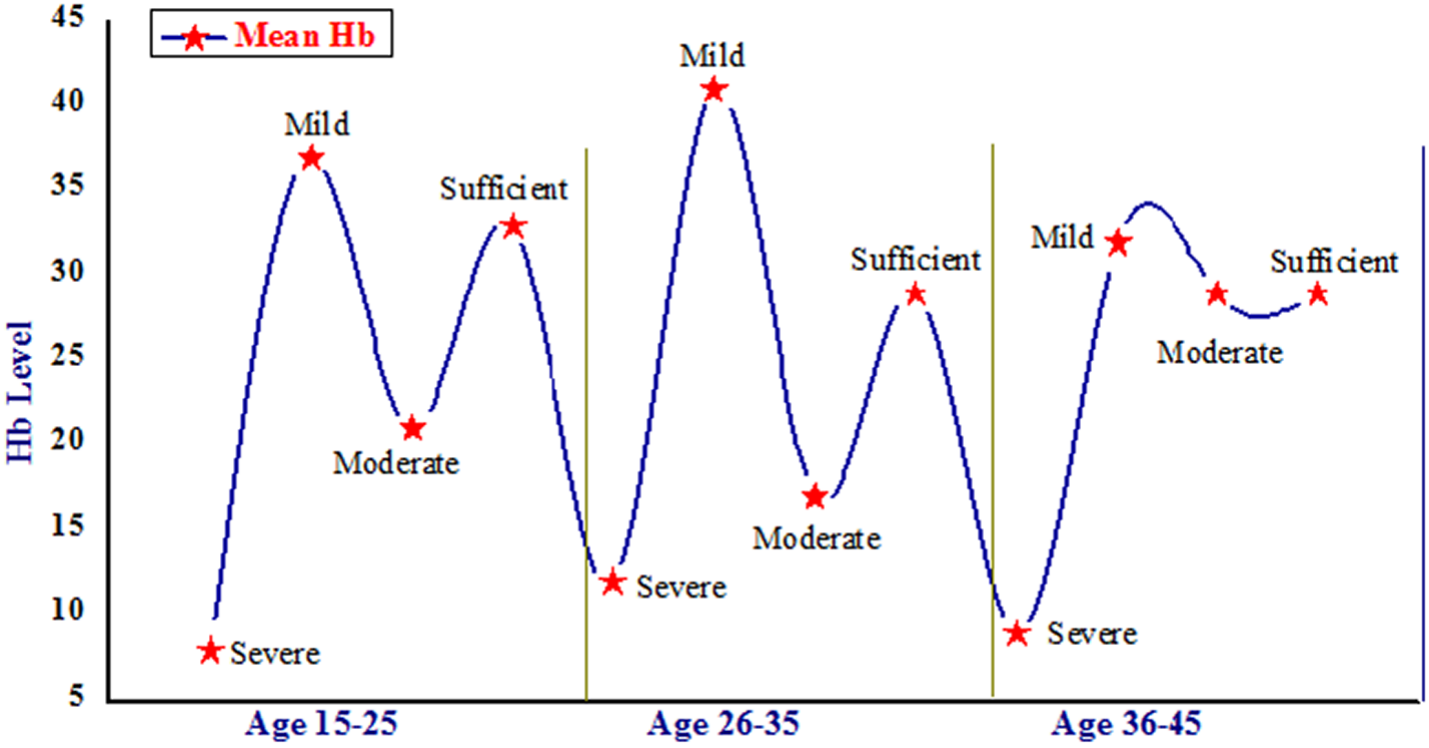
Mean Hb levels of different RAG of women.

The data regarding Hb levels in different RAG, 15–25, 26–35, and 36–45, were recorded as 10.22, 10.41, and 9.90 g/dL **(**Table 1 **)**, indicating the mild anemic situation in Gilgit district, whereas the coefficient of variation (CV) for Hb levels in different RAG was 9.64, 18.27, and 18.19% representing heterogeneity. The study region was divided into three strata, the Hb level in strata I and III delivered nonsignificant, while stratum II showed significantly different (<0.05) results among different RAG (Table 2 **)**. Moreover, the RAG 15–25 had 8% severe, 37% mild, 21% moderate, and 33% sufficient Hb levels. In RAG 26–35, 41% were mild, 17% moderate, 12% severe, and 29% sufficient in their Hb level. Furthermore, the RAG 36–45 showed 32% mild, 29% moderate, 9% severe, and 29% sufficient results (Figure 4). To characterize the Hb level relationships with different RAG, Pearson's correlation was calculated for each group. Results showed age group 15–25 had a negative correlation with the age group 26–35, whereas the age group 15–25 showed a positive correlation with the 36–45 group and the age group 26–35 presented a negative correlation with the 36–45 group **(**Table 3 **)**.

**TABLE 1:** Descriptive statistics of Hb level in different RAG of women.

RAG	Min	Max	Mean	SD	CV %	Skewness	Kurtosis
15–25	5.30	14.20	10.22	2.00	19.64	−0.18	−0.37
26–35	5.30	14.00	10.41	1.90	18.27	−0.20	−0.25
36–45	5.60	13.20	9.90	1.80	18.19	−0.03	−0.49

**TABLE 2:** Hb level of different RAG of women in different strata of Gilgit district.

RAG	Stratum I	Stratum II	Stratum III
15–25	10.51 ± 2.09^A^	9.72 ± 2.36^B^	9.75 ± 2.25^A^
26–35	9.81 ± 2.18^A^	11.22 ± 1.84^A^	10.16 ± 1.69^A^
36–45	10.34 ± 1.58^A^	10.31 ± 1.76^AB^	9.81 ± 1.40^A^
SE	0.57	0.52	0.51
CVC	1.14	1.03	1.02

*Note*: Values with the same letter in the column are statistically not different at LSD (0.05).

**TABLE 3:** Pearson correlations among women of RAG.

	Age 15–25	Age 26–35	Age 36–45	Rep
Age 26–35	−0.0703			
Age 36–45	0.0929	−0.1003		
Replication	−0.0671	0.0648	−0.1751	
Treatment	−0.0368	0.1287	0.0064	0.0400

Abbreviation: RAG, Reproductive Age Group.

### 3.2 Geostatistical analysis

**FIGURE 5:**
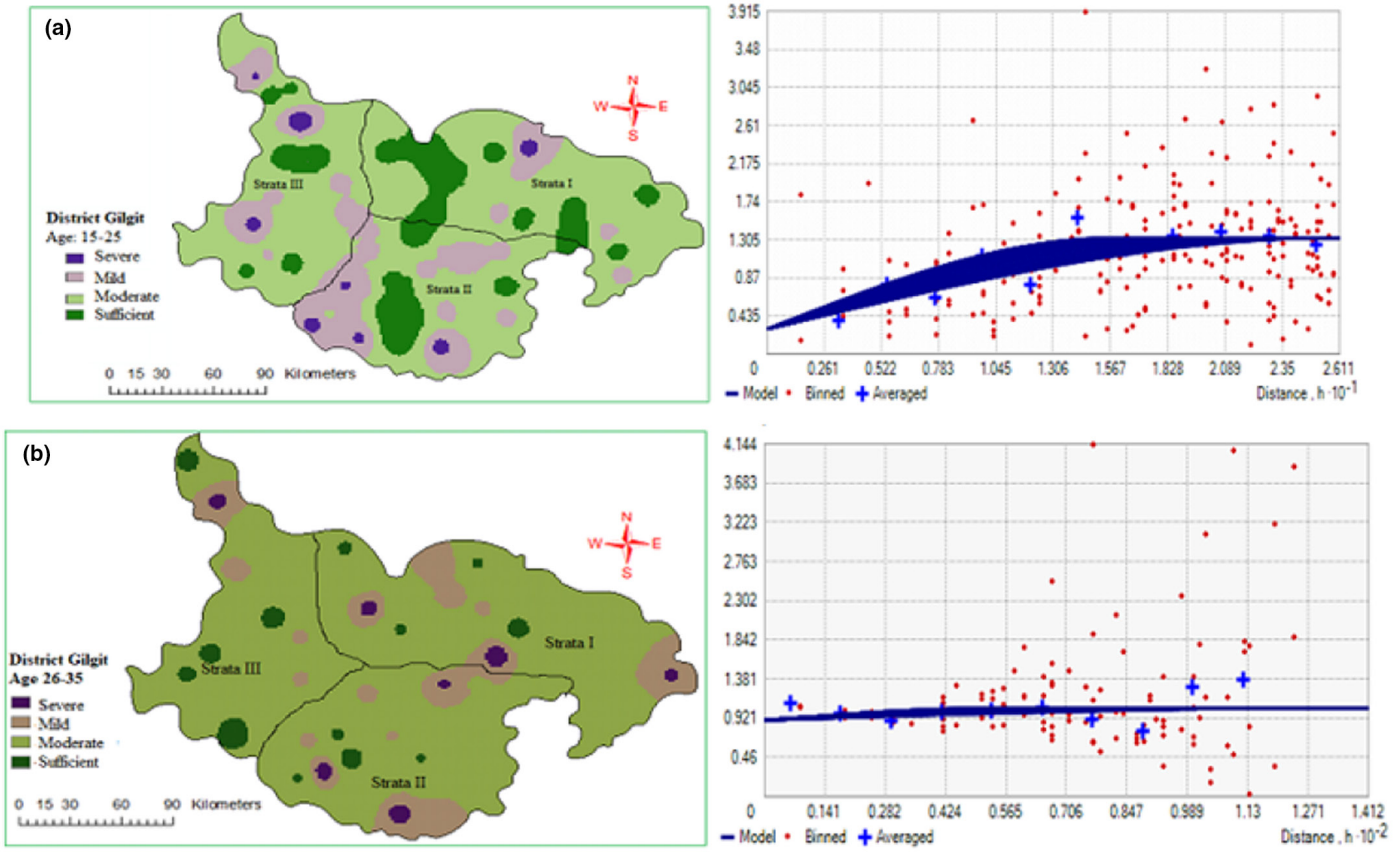
Spatial distribution map and semivariogram of Hb level at RAG of women (15–25) (a) and (26–36) (b) in Gilgit district.

**FIGURE 6:**
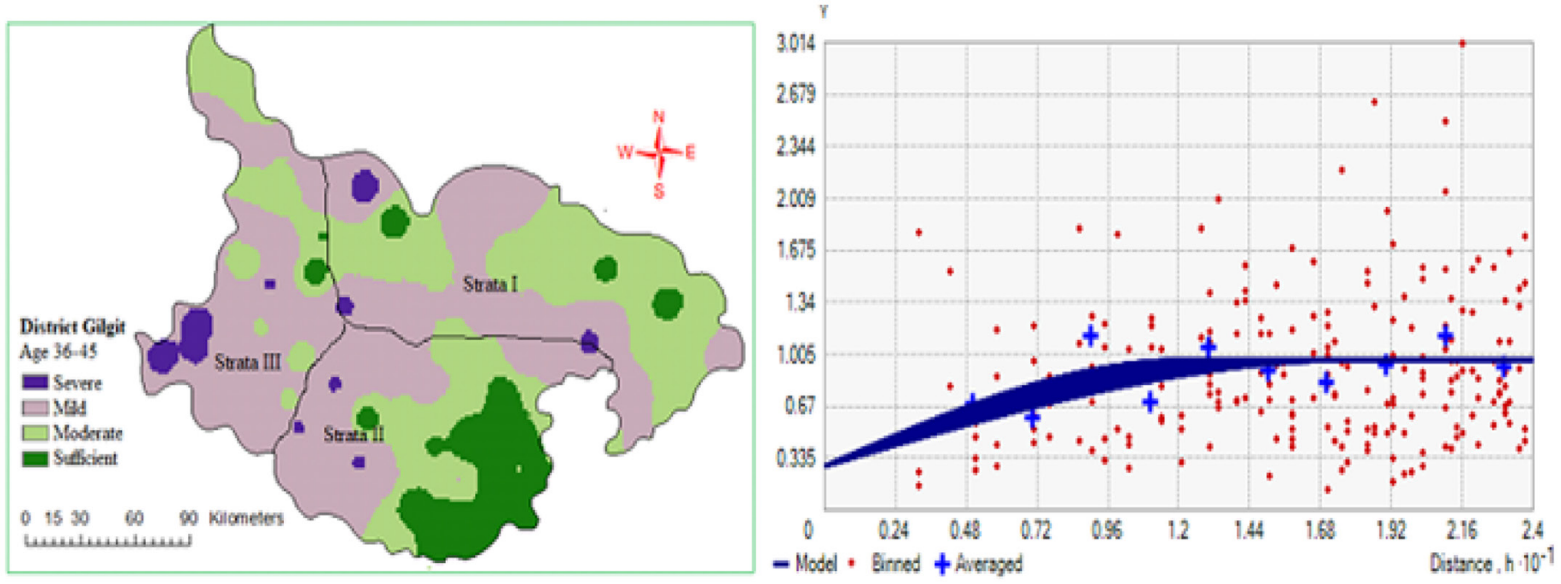
Spatial distribution map and semivariogram of Hb level at RAG of women (36–45) in Gilgit district.

Spatial variability of Hb level in women of RAG was measured by semivariogram method followed by analysis and fit into the spherical model. The nugget–sill ratio was used to assess the spatial dependence of Hb level in women of RAG. The age group 15–25 showed strong spatial dependency, while 26–35 presented weak and 36–45 had moderate spatial dependence (Table 4 **)** correlation, respectively. Nonetheless, the parameters of the spherical model were fruitful for inverse distance weight (IDW) to produce the spatial distribution maps of the Hb level of women of RAG in the study area. Maps indicate that all three strata of different RAG depicted mild‐to‐moderate Hb level outcomes with very low frequency of Hb level in Gilgit district (Figures 5 and 6).

**TABLE 4:** Geostatistical analysis of Hb level in different RAG of women in Gilgit district.

RAG	Model	Range	*N* (*C*_0_)	PS (*C*)	*S* (*C*_0_ + *C*)	*N*/*S* ratio	SDI %	Spatial dependence
15–25	Spherical	15.8	0.28	1.04	1.32	0.21	21.21	Strong
26–35	Spherical	27.12	0.89	0.14	1.03	0.86	86.40	Weak
36–45	Spherical	18.25	0.28	0.68	0.96	0.29	29.16	Moderate

*Note*: *N*/*S* ratio = [*N*/(*N* + PS)].

Abbreviations: *N*, nugget; PS, partial sill; RAG, Reproductive Age Group.

### 3.3 Determinant factors for anemia at individual level

**FIGURE 7:**
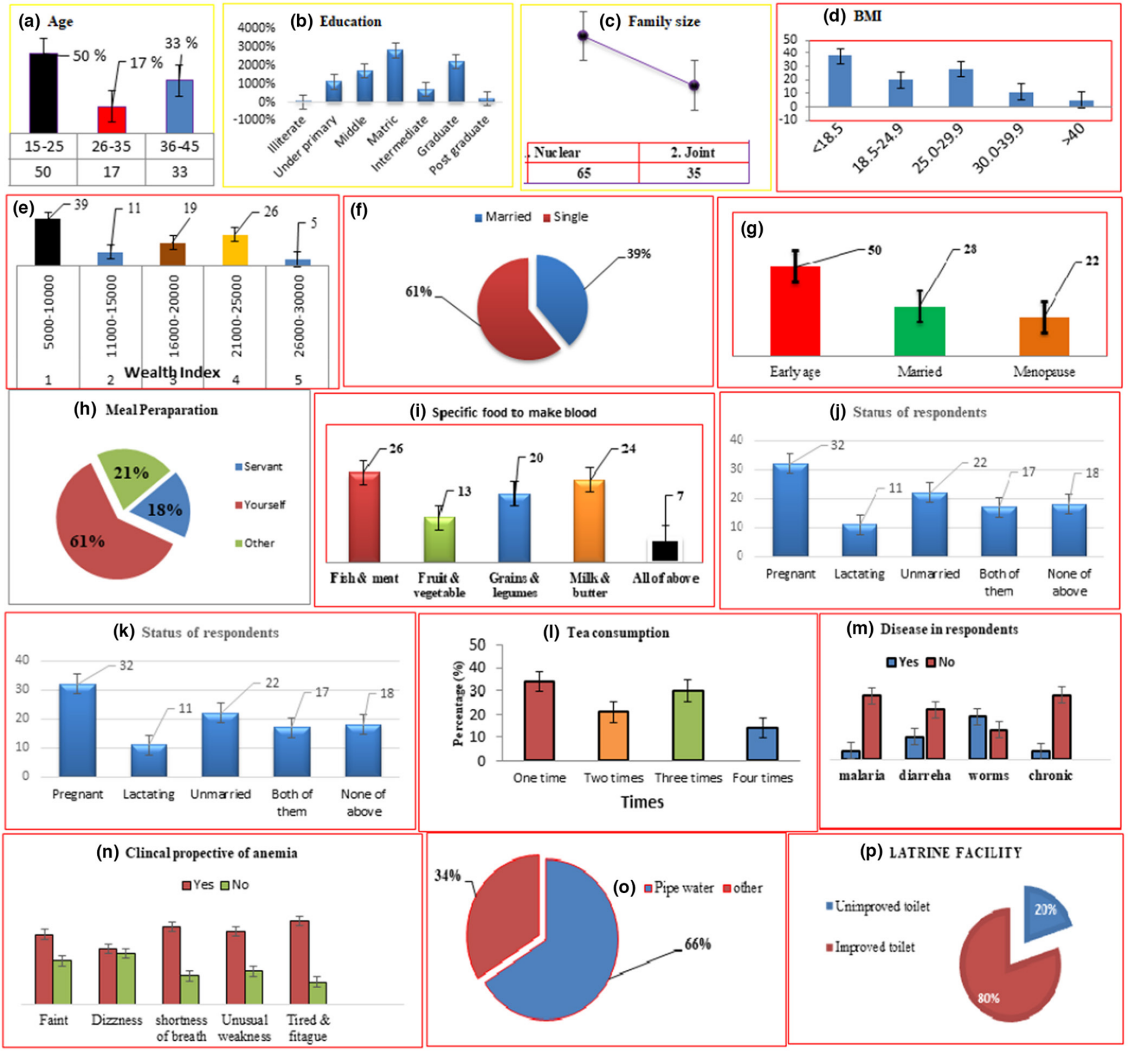
Determinant factors of anemia in Gilgit district.

Anemia is among common health problems throughout the world, especially in developing countries including Pakistan (Habib et al., 2020). The determinant factors of anemia were comprised of community and individual levels. The individual factors include age, educational status, marital status, BMI, family type, profession, wealth index development age, preparation of a meal, knowledge about balanced diet and calories, specific food responsible for blood synthesis, their status, and taking tea (Figure 7 A‐P). The respondents in the 15–25, 26–35, and 26–35 age groups consist of 50%, 17%, and 33% in the study area, respectively.

The BMI assessment showed 30% low, 20% had normal, 28% had increased BMI, 30% belongs to obesity class I, 6% was classified in obesity class II, and 5% belongs to class III. However, obesity showed a positive relation with anemia. Nonetheless, our study also established that undernourished women (BMI < 18.5) were more susceptible to anemia than others (normal and high). The outcomes are similar to other studies conducted by Vindhya et al. (2019) and Wawer et al. (2021).

Moreover, the monthly income of the respondents with 5000–10,000 (Rs) income was 39%, 11,000–15,000 earners were 11%, 19% manage 16,000–20,000, 26% earns 21,000–25,000, while 5% had 26,000–30,000 income in terms of salaries, business, agricultural income, and others. According to previous studies, people living in rural areas belonging to poor socioeconomic status and illiterate women are more prone to anemic conditions, educated women know the availability of health services and also utilize the information more efficiently (Liyew et al., 2021). In this study, there was variation in the anemia rate in terms of the educational status of the women. The anemia association with education was 13% illiterate, 2% postgraduate, 22% graduate 7% intermediate, 28% metric, 17% middle, and 11% under primary levels. The incidence of anemia increases steadily with the decrease in educational achievements. In a survey, high anemic cases were also observed among women in the poorest wealth status (Alaofè et al., 2017). This might be due to less money to buy nutritious food, which in turn leads to inadequate nutrient intake and nutritional status. A recent survey also presented that families with lower income (29.6%) had the highest anemic issues than other wealth categories (Abate et al., 2021).

In addition to other factors, marital status is also related to the frequency of anemia and Hg levels. The bereavement can result in higher levels of pro‐inflammatory interleukin‐6, one may expect that widows and widowers may have a higher risk of anemia or chronic inflammation (Moran‐Lev et al., 2018). Our survey showed 61% as married, while 39% were single in the study area. The early age was calculated as 50%, the married population was 28%, and the menopause population was 22%. Gilgit district results showed that women of reproductive age between 15 and 26 are suffering from anemia due to area (rural), unhealthy diet, lack of awareness, and menstrual blood loss which are considered the determinant factors. Furthermore, lactating mothers are more likely to have anemia than nonlactating mothers (Siddiqui et al., 2017). Similarly, according to the current status of the reproductive age women, 32% were pregnant, 11% were lactating, 17% were both, 22% were unmarried, and 18% were none of them, unfortunately, anemic cases were observed in all the age groups.

Moreover, some studies showed that older age is another important and significant determinant of anemia (Kamruzzaman et al., 2015). Likewise, women having more children were at risk of anemia as demonstrated in some research studies (Page et al., 2015). In Gilgit district, women between the age of 36 and 45 were only 2.66% with severe anemic conditions due to healthy diet, awareness, and targeted interventions. The results are similar to the results established by Kamruzzaman et al. (2015), reporting about 9.34% of anemic cases in 36–45 reproductive‐age women. The women aged 40–49 had a lower anemic as compared to women aged between 15 and 19 years. This finding is in line with early studies evaluated by Alaofè et al. (2017)).

Anemia situation was different for each developmental age of the respondents. The meal preparation assessment indicated that 18% of respondents hired servants for the preparation of their meals, 61% prepare themselves, and 21% were others. According to a diet‐related survey, 26% of women believed that fish and meat make blood, while 13% think fruits and vegetables make blood, 20% of respondents think grains and legumes make blood, 24% told that milk and butter make blood, and 7% told all food can make blood.

Likewise, iron absorption can be significantly reduced by tea consumption (Alzaheb & Al‐Amer, 2017). In 2015, Dutch dietary guidelines recommended that drinking three cups of tea daily can associate with a lower risk of stroke and diabetes. However, drinking too much green tea may lead to iron deficiency (Lazrak et al., 2021). The negative relation of tea (too much) with anemia has been also reported due to a reduction in iron absorption (Bansal et al., 2020; Feleke & Feleke, 2018), and others. Our questionnaires about tea consumption showed that 34% of respondents take tea per day, 21% consume it two times per day, 30% take it three times, and 14% take it four times per day.

Likewise, the association of certain diseases such as malaria, hookworm infestation, and chronic illness with anemia is also reported in different studies. Malaria infection in humans by Plasmodium species is associated with a reduction in Hb levels causing anemia (Rogerson, 2017). Our findings showed that 12% of respondents suffered from malaria, 31% of respondents suffered from diarrhea, 59% of respondents had intestinal worms problems, and 11% of respondents had different chronic ailments. The results were similar to the findings reported by Kibret et al. (2019) and Alaofè et al. (2017). Meanwhile, intestinal worm result was higher than those reported by Bolka and Gebremedhin (2019)) because groundwater with fecal bacteria has the direct approach to the epidemic of waterborne diseases such as diarrhea, intestinal worms, and typhoid.

Our current study supported that the people living in rural areas, poor wealth index, malaria, and worm infestation had positively associated with anemia. In addition, early studies also established that people with a history of infection cause anemia through loss of nutrients, decreasing appetite, decreasing efficiency of absorption, and use of nutrients. Higher prevalence of anemia among women with parasitic infestations due to the same line of reasoning forwarded for infection (Nadiyah et al., 2021).

Likewise, the clinical perspective showed that 21% of respondents had tiredness and fatigue symptoms, 31% suffered from unusual weakness, 27% suffered from shortness of breath, 48% were affected by dizziness, and 41% suffered from the faint. Meanwhile, after suffering from anemic symptoms, 2% of respondents took rest, 13% visited doctors, 22% visited dispensers, 42% visited Hakeem, 11% took various foods, 6% took multivitamins, and 4% include others (Shah et al., 2021). Availability of clean water and latrine facilities showed that 66% utilize improved water source, whereas 80% employ improved toilets. According to a recent report, the population having unimproved toilet facility odds of anemia. Improper latrine facilities can lead women to helminthic infections, and lead to anemic conditions (Kibret et al., 2019).

## 4 CONCLUSION

A geographical information system is useful for a diverse exploration of human health policy formulation. The current endeavor has explored geospatial distribution and determinant factors associated with anemia in women of RAG. Results showed that anemia is spatially distributed in the study area and the outcomes may be applied to site‐specific management practices to mitigate the burden. Descriptive statistics revealed that 9.38% severe, 36.89% mild, 12.88% moderate, and 30.66% sufficient cases in Gilgit district. In the near future, mild and sufficient anemia cases can turn into severe cases. So, timely interventions are needed to cope with the burden. The determinant factors were education, diet, age, BMI, latrine facilities, improved and unimproved water sources, diarrhea, intestinal worms, chronic disease, family type, and unemployment in the study. Among, education, diet, BMI, latrine facilities, unimproved water source, intestinal worms, diarrhea, chronic disease, family type, unemployment, lactating women, pregnant women, and excessive use of tea were the triggering factors. To cope with this issue, awareness sessions, workshops, and seminars must be conducted in district Gilgit.

## AUTHOR CONTRIBUTIONS

**Ghulam Abbas:** Conceptualization (equal); data curation (equal); writing – original draft (equal). **Azhar Hussain:** Conceptualization (equal); reviewing and editing (equal). **Abid Hussain:** Supervision (equal); Writing – reviewing and editing (equal). **Zahoor Ahmed:** Data curation (equal); formal analysis (equal). **Yasir Abbas:** Softwere (equal). **Arash Nemat:** Data curation (equal); writing – review and editing (equal).

## FUNDING INFORMATION

None.

## CONFLICT OF INTEREST STATEMENT

All the authors declare that they have no conflict of interest.

## ETHICAL APPROVAL

For research involving human subjects, the research project has received ethical approval from the Department of Agriculture and Food Technology at Karakoram International University, Gilgit.

## Data Availability

The dataset supporting the conclusions of this article is included within the article.
